# Structural Diversity, LC-MS-MS Analysis and Potential Biological Activities of *Brevibacillus laterosporus* Extract

**DOI:** 10.3390/metabo12111102

**Published:** 2022-11-11

**Authors:** Muhammad Zayed, Islam M. El-Garawani, Sabha M. El-Sabbagh, Bassem Amr, Sultan M. Alsharif, Ahmed A. Tayel, Mohamed F. AlAjmi, Hasnaa M. S. Ibrahim, Qiyang Shou, Shaden A. M. Khalifa, Hesham R. El-Seedi, Nora Elfeky

**Affiliations:** 1 Department of Botany and Microbiology, Faculty of Science, Menoufia University, Menoufia 32511, Egypt; 2 Department of Zoology, Faculty of Science, Menoufia University, Menoufia 32511, Egypt; 3 Biology Department, Faculty of Science, Taibah University, Al Madinah 887, Saudi Arabia; 4 Department of Fish Processing and Biotechnology, Faculty of Aquatic and Fisheries Sciences, Kafrelsheikh University, Kafrelsheikh 33516, Egypt; 5 Department of Pharmacognosy, College of Pharmacy, King Saud University, Riyadh 11451, Saudi Arabia; 6 Department of Chemistry, Faculty of Science, Menoufia University, Menoufia 32511, Egypt; 7 Second Clinical Medical College, Zhejiang Chinese Medical University, Hangzhou 310058, China; 8 Department of Molecular Biosciences, The Wenner-Gren Institute, Stockholm University, S-106 91 Stockholm, Sweden; 9 International Research Center for Food Nutrition and Safety, Jiangsu University, Zhenjiang 212013, China; 10 Pharmacognosy Group, Department of Pharmaceutical Biosciences, Uppsala University, Biomedical Centre, SE 751-24 Uppsala, Sweden; 11 International Joint Research Laboratory of Intelligent Agriculture and Agri-Products Processing, Jiangsu Education Department, Jiangsu University, Nanjing 210024, China

**Keywords:** *Brevibacillus laterosporus*, marine sediment, cyclo dipeptides, cytotoxicity, antimicrobial, phylogenetic analysis

## Abstract

Lake Mariout is Egypt’s degraded coastal marine habitat that encompasses a variety of wastes. The biodiversity and hard environmental conditions allow the co-existence of organisms with high resistance and rich metabolism, making them potential candidates for screening and isolating novel microbial strains. A bacterial isolate (BF202) cultured from the marine sediments of Alexandria’s Mariout Lake (Egypt) was tested for its antimicrobial and anticancer potential. The phylogenetic analysis of the isolated strain’s *16S rDNA* and *gyrB* revealed that BF202 belongs to *Brevibacillus laterosporus* (*B. laterosporus*). Antibiosis of *B. laterosporus* was confirmed against microbial pathogens including *Escherichia coli, Klebsiella pneumoniae*, *Salmonella typhi*, and *Staphylococcus aureus*. The highest antibacterial activity was detected on glucose peptone medium after 18 h of incubation at 35 °C, and at pH of 7.0 in the presence of mannose and ammonium carbonate as carbon and nitrogen sources, respectively. The cytotoxicity of the methanolic extract against breast cancer (MCF-7) and normal Vero cell lines, using the MTT test, revealed IC_50_ values of 7.93 and 23.79 µg/mL, respectively. To identify apoptotic and necrotic cells, a flow cytometric analysis using annexin V-FITC/PI dual-labeling was utilized and recorded a higher number of necrotic cells compared to apoptotic ones. Similarly, the cell cycle S-phase arrest was reported. The LC-MS-MS investigation of *B. laterosporus* extract and the molecular networking database analysis demonstrated five strategic diketopiperazine compounds with antimicrobial and anticancer activities. Taken together, this research shows that the crude extract of *B. laterosporus* might be an effective agent against drug-resistant bacteria and malignant disorders due to its richness in diketopiperazines.

## 1. Introduction

Antibiotics resistance is one of the most serious challenges facing today’s healthcare system, posing a serious threat to public health [1,2] because of the spread, emergence, and persistence of multidrug-resistant (MDR) bacteria. Immediate and coordinated strategies are required to avoid the lethal threat posed by antibiotic-resistant microbes. One of the actions could be finding an alternative antimicrobial agent with a new mechanism of action. Antimicrobials are drugs that impede, alleviate, or treat illnesses caused by pathogens. These drugs can be grouped into various classes according to the target pathogens, such as antibiotics, antivirals, antifungals, and antiparasitics. Antibiotics are classified into two clusters: bactericidal (killing the bacteria) or bacteriostatic (reducing the bacteria development and proliferation) [3]. Accordingly, the underlying mechanisms of action can vary from interfering with cell wall formation, protein buildup, and nucleic acid generation to disrupting membrane functions and metabolic processes [4].

Equally as interesting, bacterial infection has been claimed to contribute cancer formation via two mechanisms: either the development of chronic inflammation—for instance, *Helicobacter pylori* infestation is claimed to be a pre-carcinogenic insult to the stomach, causing stomach cancer—or the production of mutagenic bacterial metabolites. [5,6]. Although in vivo research on the carcinogenesis of bacterial metabolites is conflicting, developing a medication that has antibacterial activity and potent anti-inflammatory and antioxidant properties can be a promising solution for prophylactic and curative protocols. Microbial peptides (MPs) can be potential candidates for this purpose, especially as they have anticancer effects, according to emerging data [7]. They have shown abilities to limit malignant cell proliferation as they electrostatically adhere to cancer cells’ negatively charged outer membranes, thus producing a cytotoxic effect via necrosis or apoptosis. Additionally, it was evidenced that they have a low likelihood of acquiring resistance or causing damage to healthy cells [1,8,9,10,11].

*Brevibacillus* sp. are among the species known for their ability to produce multiple short-sequence MPs along with antimicrobial agents such as glycopeptides, brevibacillin, and bacteriocin [12]. For example, brevilaterin B and other peptides extracted from *Brevibacillus laterosporus* S62-9 were recognized for their anticancer, antibacterial, and antifungal activities [13]. Similar effects were generated by *Brevibacillus brevis* EGS9 strain, as reported previously [14]. Moreover, bogorol, a peptide isolated from *Brevibacillus laterosporus* JX-5, exhibited potent antibacterial and anticancer activities [15]. *Brevibacillus* was described as a facultatively anaerobic, Gram-positive, and endospore-forming bacterium [16], and thus was further reclassified from *Bacillus* [17]. *Brevibacillus* can be found in various environments, including oceans, rivers, sediment, hot springs, soil, and compost [18].

Microorganisms, especially halophilic or halotolerant bacteria, from severe environments have recently received much attention for their ability to produce biosurfactants, enzymes, and antimicrobial agents as a tool to survive and defend their existence [19,20,21,22,23]. Some of these severe saline environments occur in Egypt, namely the lakes of Wadi Al-Natrun [24], the Solar Lake on the Sinai coast of the Gulf of Aqaba [25], and Mariout Lake in the city of Alexandria [26]. Mariout Lake is one of the Nile Delta’s most anthropogenically degraded and eutrophic wetlands due to outflows from the industrial and urban sectors [27]. The BF202 strain was distinguished from the early reported strains, i.e., JX-5 (GenBank: KF444391.1) and S62-9 (GenBank: EU709016.1), with the similarity presented as 99.22% and 99.39%, respectively. The 16S rRNA identities were applied and supported the notion that the alteration of the genetic structure could be ascribed to the effect of environmental factors [15,28].

We aimed to isolate halophilic and halotolerant bacteria from the hypersaline Mariout Lake in order to characterize the marine *B. laterosporus* BF202 and scrutinize their bioactivities. More attention was paid to the *B. laterosporus* BF202 isolated bioactive metabolites that could function as promising antibacterial and anticancer leads. To our knowledge, using LC-MS-MS analysis, identifying certain diketopiperazines, and investigating anticancer and antibacterial potentials in the crude extract of marine *B. laterosporus* of Egypt has not been realized before, and this is the first publication on this point in parallel to our continuous and ongoing interest to explore the chemical and biological features of natural organisms [29,30,31,32].

## 2. Materials and Methods

### 2.1. Isolation and Cultivation Conditions of Halotolerant Bacteria

Twenty soil sediment samples were collected from Mariout Lake (Alexandria, Egypt) at a depth of 30 cm and stored in a sterile polyethene packet at 4 °C. Five grams of each sediment sample were serially diluted with sterilized seawater. Each dilution was then spread onto a Petri dish containing tryptone soya agar (TSA) in seawater. The plates were kept for 48 h at 37 °C. The plates with 20–200 colonies were selected for further processing. The bacterial colonies were picked and streaked on TSA plates to obtain purified bacterial colonies. 

### 2.2. Antibacterial Potencies against Pathogenic Bacteria

#### 2.2.1. Paper Disc Assay 

Using the paper disc assay, twenty isolates were screened for their antimicrobial potencies towards four microbial pathogens, namely *E. coli* ATCC 8739, *S. typhi* ATCC 14028 *S. aureus* ATCC 6598, and *K. pneumoniae*. A flask containing 50 mL of tryptone soya broth (pH 7.2) and about 1 mL (1.5 × 10^8^ cfu/mL) of the purified bacteria was inoculated and incubated overnight at 100 rpm and 37 °C (Precise Shaking Incubator, WIS-RL010, Ulm, Germany). Following the incubation period, growing bacteria were collected at 3000 rpm for 30 min. A 0.45 µm bacterial filter was used to filter and sterilize the supernatant. The culture filtrate was used to saturate the filter paper disc (5 mm in diameter) to test its antagonistic activity. Then, the discs were placed gently on previously streaked plates by pathogenic bacteria. After incubation for 24 h at 37 °C, the inhibition zones’ diameters were taken. The most active isolates were chosen for further investigation.

#### 2.2.2. Agar Well Diffusion Assay

At room temperature, 0.5 mL (1.5 × 10^8^ cfu/mL) of fresh pathogenic bacteria spore suspensions was mixed with 9.5 mL of melting sterile Muller Hinton agar (45 °C), then poured onto sterile Petri dishes and allowed to solidify. A sterile cork borer with a 7 mm diameter was used to make wells in the inoculated agar plates. Each well was filled with 100 µL of the previously prepared sterile cell-free culture supernatant. The inhibition zones’ diameters were recorded after incubation for 24 h at 37 °C (Binder BD53, Tuttlingen, Germany) [33].

### 2.3. Molecular Identification of the Isolated Bacteria

The molecular identification of the isolated bacterium was performed through Macrogen, Korea. DNA extraction and purification were performed via InstaGene Matrix (BIO-RAD, cat. no. 732-6030), as described by the supplier’s protocol. Purified DNA was considered pure when the OD_260_/OD_280_ ratio was around 1.8 and the OD_260_/OD_230_ ratio was about 2.0. To sequence the 16S RNA encoding gene, the polymerase chain reaction (PCR) was performed using the Taq polymerase Dr. MAX DNA Polymerase (Doctor Protein, Korea, cat. no. DR00302) and the universal primers 27F/1492R through the DNA Engine Tetrad 2 Peltier Thermal cycler (Applied Biosystems, Foster City, CA, USA). The PCR product was purified through the Multiscreen Filter Plate (Millipore Corp., Darmstadt, Germany). Afterwards, the sequencing was performed using Sanger (dideoxy) technology via the sequencing kit BigDye (R) Terminator v3.1 Cycle Sequencing Kit (Applied Biosystems, Vilnius, Lithuania) and using the universal primers 785F/907R. The sequencer ABI PRISM 3730XL Analyzer (96 capillary type) was used. After assembling the sequencing and removing the low-quality regions, the sequence was deposited in the nucleotide database of the National Center for Biotechnology Information [34]; the sequence identifier is OP03. However, it was not sufficient to discriminate *Brevibacillus laterosporus* from *Brevibacillus halotolerans*. The DNA gyrase subunit B was amplified through the thermal gradient PCR and using the forward primer 5′-CGAGCACTATCATATACCGAAGA-3′ and the reverse primer 5′-GGACTTTATCGCCCATTAAAACC-3′. The PCR thermal gradient program was a single cycle of 95 °C for 5 min and 30 s, followed by 35 cycles at 95 °C for 30 s, 48–68 °C for 30 s, 72 °C for 1 min, and an elongation step at 72 °C for 7 min. The gel electrophoresis was seen on a 1.5% agarose gel, 20 min running at 300 V, 200 A, and 2 µL of each PCR product was loaded into each well. Additionally, 2 µL of the GeneRuler DNA ladder Mix (#SM0331, Applied Biosystems, Vilnius, Lithuania) was loaded. Finally, the product of the annealing temperature of 60 °C was selected as the best and was sequenced with the same PCR primers. The obtained sequences were assembled into contig, and the low-quality regions were removed. The generated sequence was deposited into GenBank under the accession number OP105159. 

To construct the phylogenetic trees, each sequence was used as a query in the BLASTN algorithm with a cutoff e-value of 1e−11 to retrieve the authentic homologous genes [35,36]. Retrieved sequences were aligned using MAFFT version 7 [37] (https://mafft.cbrc.jp/alignment/server/, accessed on 10 November 2022). A maximum likelihood phylogenetic tree was inferred assuming the K2P + G4 substitution model with 1000 bootstrap replicates for the *16S DNA* homologous sequences and the SYM + R4 substitution model with 1000 bootstrap replicates for the DNA gyrase subunit B homologous sequences [38,39,40]. The downloaded phylogenetic trees were visualized and presented using the iTOL (Interactive Tree of Life) webserver [41].

### 2.4. Optimizing Culture Conditions of the B. laterosporus BF202 Antibacterial Activity

#### 2.4.1. Different Types of Growth Media

The best medium for *B. Laterosporus* antibacterial activity was determined using various growth media. In Erlenmeyer flasks (250 mL) containing 48 mL of the studied growth medium, 2 mL of bacterium suspension (1.5 × 10^8^ cfu/mL) was inoculated. The culture media used were glucose peptone medium [42], glycerol yeast extract [43], nutrient broth, and tryptic soy broth (dehydrated prepared media from HiMedia, India). The flasks were shaken on a rotary shaker (200 rpm/24 h/37 °C). All experiments were performed in triplicate. After incubation, each culture was centrifuged at 3000 rpm for 30 min (Centrifuge; Sigma 2-16P, Osterode, Germany), and the supernatants were used separately to evaluate the bacterium’s antimicrobial activity against *S. aureus* using the agar well diffusion technique.

#### 2.4.2. Different Incubation Periods 

In each 250 mL Erlenmeyer flask containing 48 mL of glucose peptone growth medium, a 2 mL inoculum of *B. laterosporus* (1.5108 cfu/mL) was cultured and incubated for different durations (18 to 72 h) under the same conditions as indicated previously. The agar well diffusion test was also used to assess the antibacterial activity of the cultures against *S. aureus*.

#### 2.4.3. Different pH Values 

The influence of various hydrogen ion concentrations on the antibacterial potency of the tested bacterium was investigated via the glucose peptone medium. Citrate–phosphate buffer pH (4.0 and 5.0), phosphate buffer pH (6.0 and 7.0), and Tris HCl buffer, pH 8.0, were used. The antibacterial activities were measured at pH 9.0 after incubation in a rotating shaker (37 °C/200 rpm/18 h). 

#### 2.4.4. Different Temperature Degrees

The organism was inoculated in glucose peptone medium (pH 7.0) to determine the optimal temperature for the tested bacterium’s antibacterial activity. It was then incubated at 200 rpm for 18 h under different temperatures, i.e., 15, 20, 25, 30, 35, and 40 °C. The antibacterial activities were determined in the manner described above. 

#### 2.4.5. Miscellaneous Carbon and Nitrogen Sources

To analyze the influence of various carbon and nitrogen sources on the antibacterial activity of *B. laterosporus*, the carbon source of the glucose peptone growth medium was replaced by an equal amount of either glycerol, sucrose, mannose, or maltose. In addition, one of the nitrogen-containing compounds (KNO_3_, NaNO_3_, NH_4_Cl, and (NH_4_)_2_CO_3_) was used to replace peptone in the peptone–glucose medium (using mannose instead of glucose). The equimolecular weight of each nitrogen compound was also considered. The antibacterial activities were determined as previously described.

### 2.5. Method for Preparing the Crude Extract

The isolated bacterium was grown on glucose peptone medium (GPM) in an incubator (100 rpm/35 °C /18 h). The supernatant was collected through centrifugation (6000 rpm/20 min), then filtered via a 0.45 μm bacterial filter. The filtrate was diluted by methanol (1:1), stirred (130 rpm/4 h), centrifuged (3000 rpm/10 min), and then evaporated through a vacuum rotary evaporator (Rotavapor, Heidolph, Schwabach, Germany). The crude extract was kept at −80 °C for future studies.

### 2.6. Characterization of Methanol Extract Using LC-MS/MS and GNPS 

For LC-MS analysis, the powder extract was dissolved in 50% acetonitrile (ACN) and 0.1% formic acid (FA). The samples were injected with a syringe through a 0.3 µL/min PicoTip emitter connected to a QTof Micro (Waters, Milford, MS, USA) with the voltage set at 1.4 kV, positive ions and a linear gradient from 10% (*v*/*v*) H2O to 99% (*v*/*v*) ACN in 0.1% (*v*/*v*) FA at a flow rate of 0.3 µL/min for 75 min, followed by LC analysis/MS-MS dissolved by 0.1 mg/mL solvent LC-MS: 60% MeCN at 0.1 with a linear gradient of 10–60% (*v*/*v*) MeCN in 0.1% (*v*/*v*) FA at a flow rate of 0.3 mL/min for 75 min. The spray voltage was set to 4 kV and the capillary temperature was raised to 220 °C [44]. The raw MS file was analyzed using Global Natural Products (GNPS) Social Molecular Networking datasets [44,45]. GNPS aids in the identification and discovery of metabolites by comparing the matching between the fragmentation pattern from the raw mass spectrum and the GNPS library. To work with GNPS, we used other installed programs such as MSConvert, File Zilla, and Cytoscape version 3.5.1. The QTOF-(+)MS/MS data were acquired at a fixed collision energy of 1.4 kV, converted from Agilent MassHunter data files to mzXML file format using MSConvert, and then transferred to the GNPS server (https://gnps.ucsd.edu/, accessed on 5 December 2021) [46]. Molecular networking was accomplished using the GNPS data analysis workflow using the spectral clustering algorithm, a minimum of 6 matched peaks, and a cosine score of 0.7. Cytoscape version 3.5.1 was used to visualize the resulting spectral networks (https://cytoscape.org/, accessed on 10 December 2021) [47]. The confirmation of these metabolites was carried out using the Dictionary of Natural Products website and the literature search conducted using Sci-Finder, PubMed, and Google Scholar.

### 2.7. In Vitro Anticancer Study

#### 2.7.1. Maintenance of Cell Lines

The Holding Company for Biological Products & Vaccines (VACSERA), Giza, Egypt, provided human breast adenocarcinoma (MCF-7) and normal African green monkey kidney (Vero) cell lines. A hemocytometer was used to determine the cell concentration per milliliter, which was then calculated using the following equation:

Cells/mL = 10^4^ × (Average count per square) × (Dilution factor)

Dulbecco’s modified eagle medium (DMEM) supplemented with 10% fetal calf serum, 100 U/mL penicillin, and 100 µg/mL streptomycin was used to maintain and culture the cell lines. Cells were cultured in T25 culture flasks at a density of 2 × 10^4^ cells/cm^2^ in a humidified 5% CO_2_ atmosphere at 37 °C. Every 48 h, the medium was changed. An inverted microscope confirmed that the cells were 75% confluent. After trypsinization (0.025% trypsin and 0.02% EDTA), cells were harvested and washed twice with phosphate-buffered saline (PBS) [48]. All experiments were carried out in triplicate. All reagents and media were obtained from Lonza (Cairo, Egypt).

#### 2.7.2. Cytotoxicity Assay 

The cytotoxicity of crude bacterial extract was evaluated on MCF-7 cells and Vero normal green monkey kidney cells using 3-(4,5-dimethylthiazol-2-yl)-2,5-diphenyltetrazolium bromide dye (MTT). Briefly, the extract was tested 24 h after incubation with a series of extract dilutions. Cells were seeded at a density of 1 × 10^4^ cells/well in a 96-well plate at 37 °C for 48 h in a 5% CO_2_ incubator and humidified atmosphere in a sterile environment until they reached 70% confluent monolayer. After 24 h of treatment, each well received 100 μL of MTT dye and was incubated for 4 h. Cells were washed with 100 μL PBS before being placed in a microtiter shaker with 100 μL of MTT de-staining solution (acidified isopropanol) for at least 10 min. To determine the number of viable cells, the optical density was measured using a microplate reader (RADIM SEAC Sirio S, Pomezia, Italy). The inhibition percentage was calculated as follows:

% Cell inhibition = (1−OD (absorbance) Test/OD Control) ×100

GraphPad Prism software (San Diego, CA, USA) was used to perform the inhibition curve and calculate the 50% maximal inhibitory concentration (IC_50_). 

#### 2.7.3. Characterization of Apoptosis Using Annexin V/Propidium Iodide (PI) Labeling

Apoptosis and necrosis were observed in the control MCF-7 cells versus the treated ones using flow cytometry via the Annexin V-FITC Kit (BD PharmingenTM, San Diego, CA, USA). Cells were collected, washed in PBS, then labeled with FITC-conjugated Annexin V and PI (Invitrogen, Carlsbad, CA, USA). The samples were analyzed with the BD AccuriTM C6 flow cytometer (San Jose, CA, USA). 

#### 2.7.4. Cell Cycle Analysis Using Flow Cytometer

A flow cytometer was used to investigate the effect of crude bacterial extract on the phases of the MCF-7 cell cycle. Cells rinsed with ice-cold PBS were fixed with ethanol, then stained in 1 mg/mL propidium iodide (PI) and 200 µg/mL RNase A-containing PBS for 15 min. The MODFIT DNA (Verity Software House, Topsham, ME, USA, version: 2.0) program determined the cell percentages in different phases. A flow cytometer (BD AccuriTM C6, San Jose, CA, USA) was employed for examining phase distribution. 

## 3. Results

### 3.1. Isolation of Halotolerant Bacteria and the Estimation of Their Antibacterial Activities

A total of 20 bacterial samples were collected from various ecological saline habitats and isolated from soil sediment. One of the isolates demonstrated significant antibacterial activity against *E. coli*, *K. pneumoniae*, *S. typhi*, and *S. aureus*, as demonstrated by the filter paper disc assay. The inhibition zone diameters were sized 21, 20, 20, and 19 mm, respectively (Table 1). Moreover, the agar well diffusion method on Muller Hinton (MH) agar was utilized to confirm the results. The isolate’s filtrate inhibited the pathogenic bacteria, namely *E. coli* (23 mm), *S. typhi* (22 mm), *K. pneumoniae* (19 mm), and *S. aureus* (20 mm), at a rate close to the standard conventional antibiotic levofloxacin (50 mg/L), as shown in (Table 2).

### 3.2. Molecular Identification of the Bacterial Isolate

**Figure 1:**
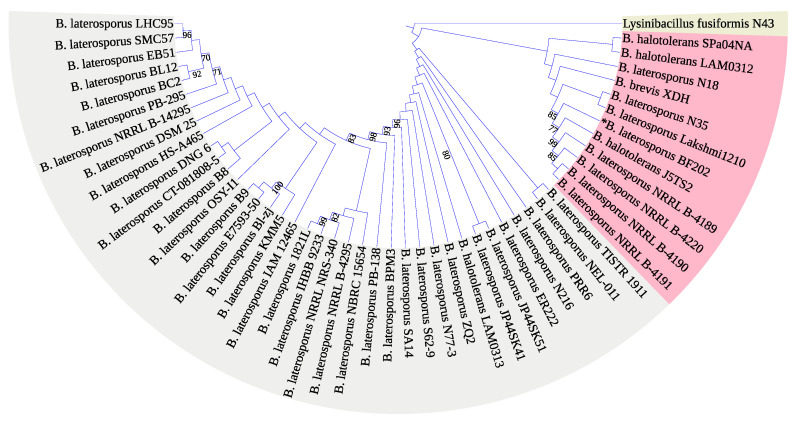
A maximum likelihood phylogenetic tree constructed based on the *16S rDNA* sequences (V_1–9_) from various bacterial strains. The homologous sequence from *Lysinibacillus fusiformis* N43 was used to root the tree.

**Figure 2:**
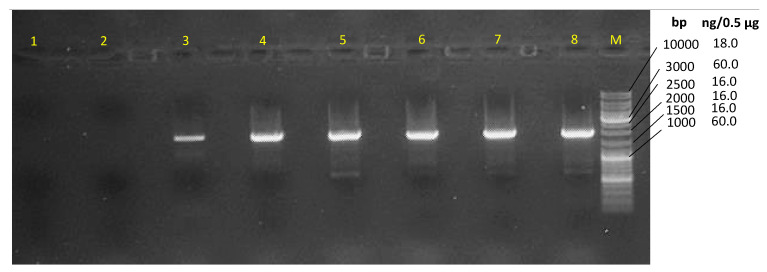
An agarose gel (1.5%) of the gradient PCR, where the annealing temperatures were lane 1, 68 °C; lane 2, 66.5 °C; lane 3, 63.9 °C; lane 4, 60 °C; lane 5, 55.5 °C; lane 6, 51.8 °C; lane 7, 49.4 °C; and lane 8, 48 °C. Marker (M), Thermo Scientific GeneRuler DNA ladder Mix (#SM0331).

**Figure 3:**
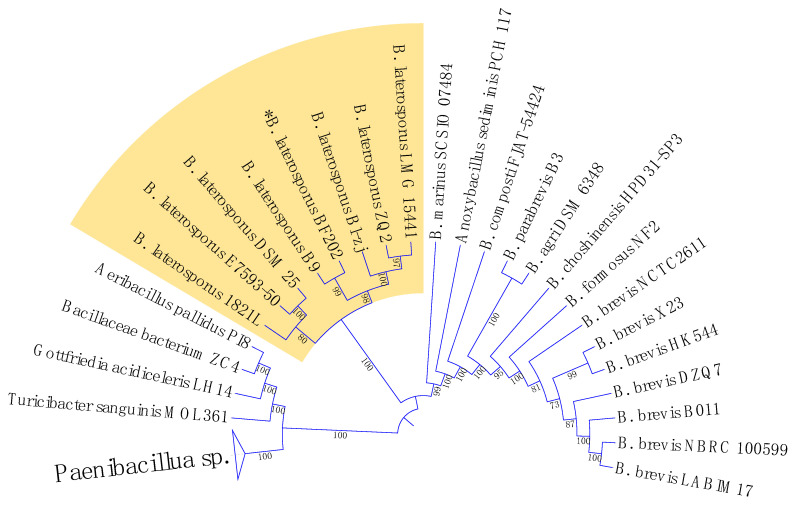
A maximum likelihood phylogenetic tree constructed based on the DNA gyrase subunit B (*gyrB*) gene sequences from various bacterial strains.

The 16S rRNA encoding gene V_1-9_ sequence was not enough to identify our isolate *Brevibacillus laterosporus* BF202 because it does not distinguish it well from the *Brevibacillus halotolerans* J5TS2 (Figure 1). March et al. [49] reported that 16S DNA is not satisfactory in separating *B. laterosporus* from other closely related species. Hereafter, the DNA gyrase subunit B (gyrB) gene was amplified through the gradient PCR (Figure 2), and its sequence was satisfactory to separate all the *B. laterosporus* strains into a distinct clade that is adequately separated from the other *Brevibacillus* species (Figure 3). The results also showed that the closest type of strain to our isolate is *Brevibacillus laterosporus* DSM 25, as indicated in Figure 3.

### 3.3. Optimization of the Culture Conditions of the B. laterosporus BF202 

**Figure 4:**
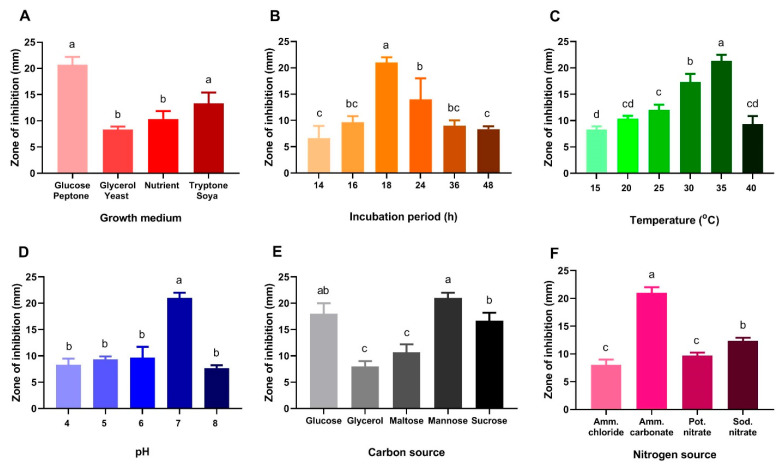
Effect of different growth conditions on the antibacterial activity of *B. laterosporus* bioactive extract; (**A**) different growth media, (**B**) different incubation periods, (**C**) different temperatures in the GPM, (**D**) different pH levels and (**E**) different carbon sources, (**F**) different nitrogen sources. The significance among different conditions was measured by Tukey’s honest significant difference (Tukey’s HSD, *p* < 0.05) using Agricolae package in R language [50,51]. Identical letters indicate that the difference is not statistically significant.

The antibacterial activity of *B. laterosporus* was studied under various culture media, mainly different incubation times, temperatures, pH levels, and carbon and nitrogen sources. Comparing different culture media, glucose peptone medium was found to be the most suitable growth medium for *B. laterosporus* (Figure 4A). As a result, the glucose peptone medium was used to optimize the other parameters. Then, the effect of different incubation times on the antibacterial activity of *B. Laterosporus* was scrutinized. The highest activity was observed after 18 h, while an additional 48 h incubation reduced the antimicrobial activity of *B. Laterosporus* filtrate by approximately 50% (Figure 4B). 

The effect of different incubation temperatures on antibacterial activity was also studied. The results showed that raising the temperature to 35 °C increased antibacterial activity by more than 50% compared to 15 °C (Figure 4C), while raising the temperature to 40 °C reduced the inhibition zone by half. The same was noticed with the pH values, so pH of 7 was selected (Figure 4D). 

In accordance with our measurements, mannose was the best carbon source, followed by glucose and sucrose. However, the lowest antibacterial activity was reported once glycerol was provided as a carbon source (Figure 4E). In regard to nitrogen sources, ammonium carbonate significantly enhanced the antimicrobial activity of the tested bacterial filtrate against *S. aureus* (Figure 4F).

### 3.4. LC-MS/MS Analysis of B. laterosporus Bioactive Extract

**Figure 5:**
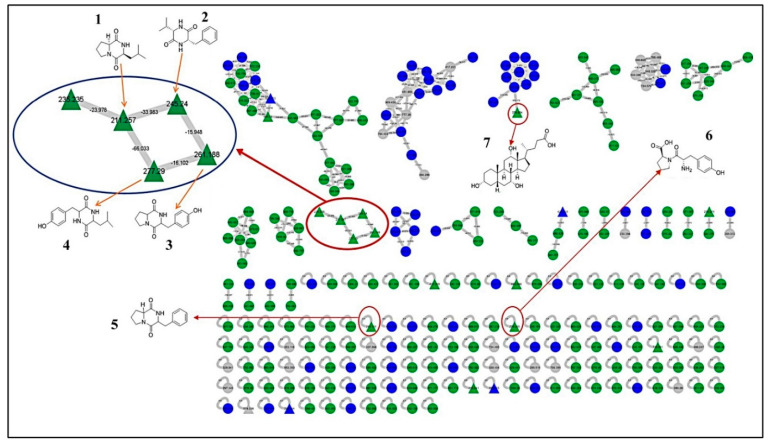
Molecular network of 165 metabolites of *B. laterosporus* MeOH extract and the blank. The gray nodes refer to the blank. The green nodes refer to the metabolites from the *B. laterosporus* MeOH extract. Blue nodes refer to the similar metabolites between the extract and blank. All the metabolites identified by name took a triangle shape.

**Figure 6:**
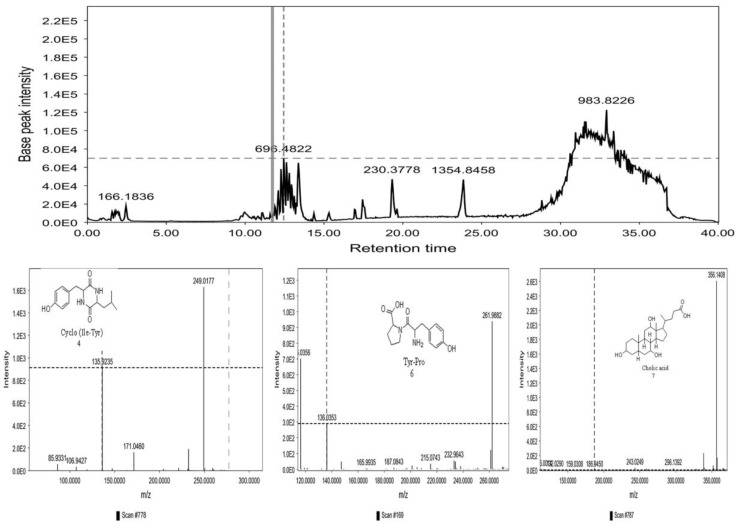
Schematic figure prescribing a base peak chromatogram of *B. laterosporus* and fragmentation of Cyclo (Ile-Tyr) (**4**), Tyr-Pro (**6**), and cholic acid (**7**).

**Figure 7:**
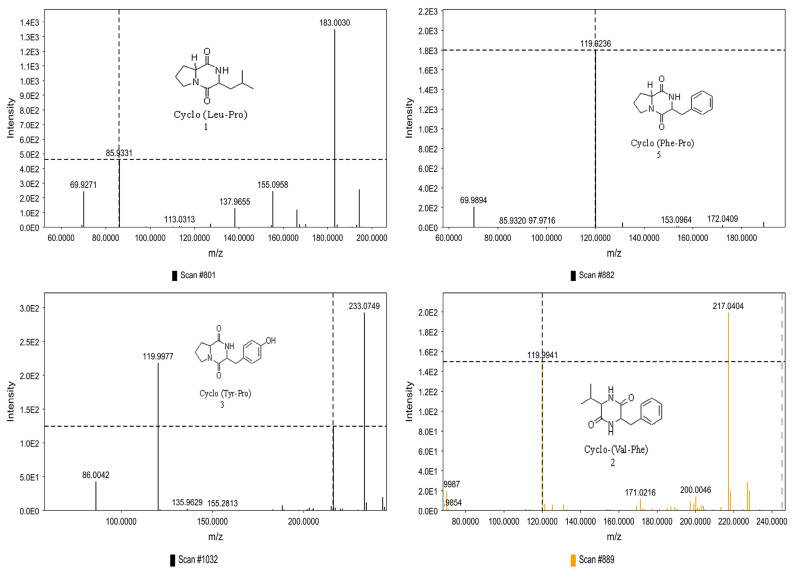
Schematic figure of MS2 spectrum of *B. laterosporus* and fragmentation of Cyclo (Leu-Pro) (**1**), Cyclo-(Val-Phe) (**2**), Cyclo (Tyr-Pro) (**3**), and Cyclo (Phe-Pro) (**5**).

The GNPS molecular networking database was used to analyze the mass metabolites of *B. laterosporus* methanol extract. A total of 165 metabolites with their masses are shown as nodes in Figure 5. One metabolite is correspondent to each node. Several metabolites from similar classes are grouped together to form a single cluster along with their associated molecular weights. Names, parent masses, and fragments of identified metabolites are shown in Table 3. Five identified metabolites were classified as diketopiperazines and were named Cyclo (Leu-Pro), Cyclo (Val-Phe), Cyclo (Tyr-Pro), Cyclo (Ile-Tyr), Cyclo (Phe-Pro), and Tyr-Pro. Cyclo (Leu-Pro), Cyclo (Tyr-Pro), and Cyclo (Tyr-Ile), and were found in the same cluster as parent masses [M^+^H]^+^ (*m/z*; 211.257), [M^−^H_2_O] + (*m/z*; 261.188), and [M^+^H^−^H_2_O]^+^ (*m/z*; 277.29), respectively (Figure 6). Another Cyclo (Phe-Pro) node was noticed with the parent mass [M^+^H^−^H_2_O]^+^ (*m/z*; 245.131) (Figure 7). In general, the identified metabolites have previously been reported to be promising antimicrobial and anti-tumor agents [52].

### 3.5. Cytotoxicity of the B. laterosporus Crude Extract

**Figure 8:**
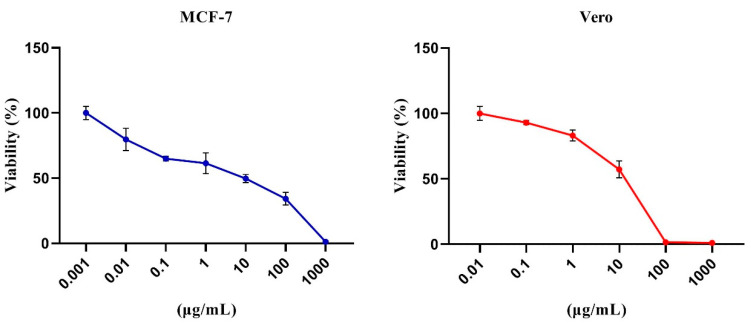
The viability of control and extract-exposed MCF-7 and Vero cell lines were tested using the MTT assay. The incubation with serial concentrations (log concentration 0–1000 µg/mL) was performed in triplicate. Data are illustrated as (mean ± SD) of three separate experiments.

The MTT assay was used to determine the cytotoxicity of the bacterial crude extract against MCF-7 and Vero cells. The extract was cytotoxic to cancer cells (MCF-7) with a mean IC_50_ of 7.93 µg/mL. In contrast, cytotoxicity was minimum when tested on Vero normal cells, with a mean IC_50_ of 23.79 µg/mL (Figure 8).

### 3.6. Quantification of Apoptosis

**Figure 9:**
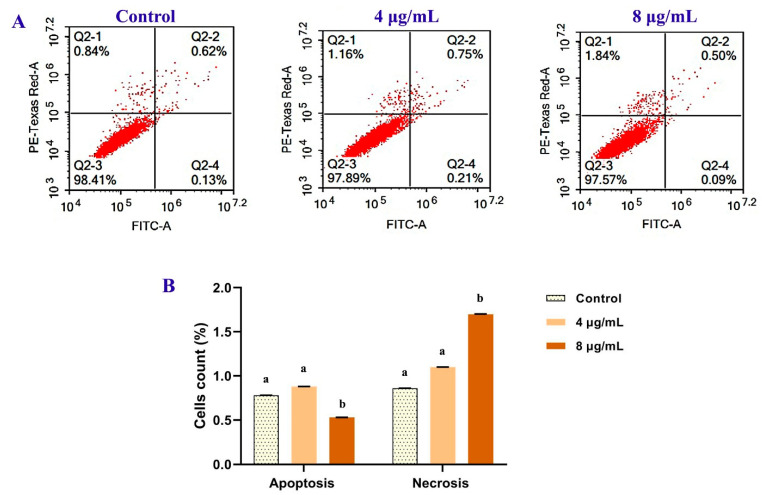
The antitumor effect of *B. laterosporus* bioactive extract (4 and 8 µg/mL) on MCF 7 after 72 h of the treatments. The flow cytometric quantification of apoptosis was performed using Annexin V-FITC/PI assay and PI staining. (**A**) Flow cytometric dot plot presentation shows the percentages of apoptotic and necrotic events. (**B**) The statistical data of treated and control groups of apoptosis/necrosis. All values are presented as mean ± SD (*n* = 3). Significant differences (*p* < 0.05) between groups are shown by different letters.

Annexin V-FITC/PI dual labeling was used to detect apoptotic and necrotic cells (Figure 7). The extent of normal, total apoptotic, and necrotic cells was evaluated as the percentage of (Annexin V−/PI−), (Annexin V+/PI−) + (Annexin V+/PI+) and (Annexin V−/PI+) cells, respectively. In comparison to control records, higher concentrations lead to a significant increase in necrotic cells (27% and 97% for 4 and 8 µg/mL, respectively), whereas apoptotic cells did not change significantly among the extract incubation (Figure 9).

### 3.7. Cell Cycle Analysis Using Flow Cytometer

**Figure 10:**
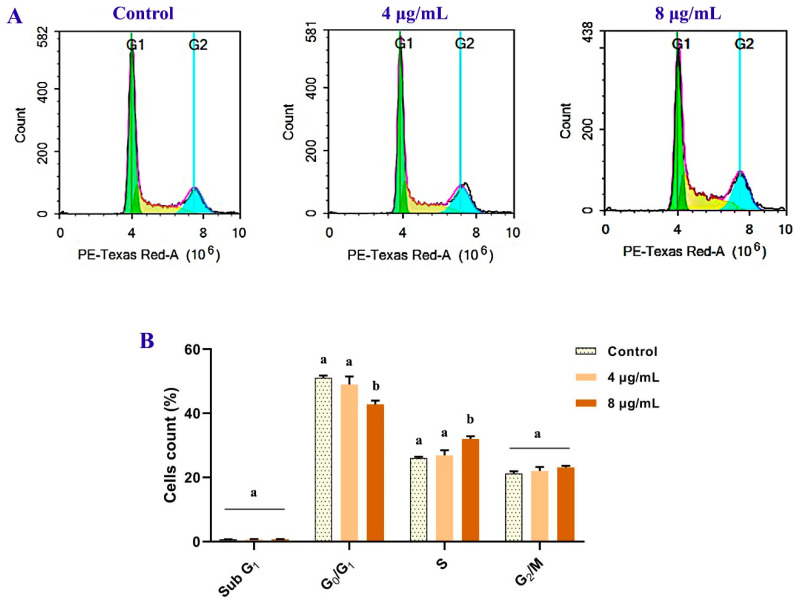
The anticancer effect of *B. laterosporus* BF202 (4 and 8 µg/mL) on MCF 7 after 72 h of the treatments. The flow cytometric quantification of the cell cycle distribution was performed using PI staining. (**A**) Flow cytometric histograms show the distribution of the cell cycle phases. (**B**) The statistical data of treated and control groups of cell cycle phases. All values are presented as mean ± SD (*n* = 3). Significant differences (*p* < 0.05) between groups at the same phase are shown by different letters.

Staining with PI and flow cytometric analysis were used to assess the effect of the extract on the MCF-7 cell cycle distribution (Figure 10). After 72 h of treatment, the percentage of MCF-7 cells in the S-phase increased from 26.01 ± 0.44% (control group) to 26.89 ± 1.59 and 32.04 ± 0.83% corresponding to doses of 4 and 8 µg/mL, respectively. Only among the higher concentrations, the records significantly (*p* < 0.05) increased. However, the percentage of G_2_/M increased non-significantly, which is associated with a marked decrease in the G_0_/G_1_ phase, indicating that *B. laterosporus* extract inhibited the cell cycle in the S-phase. These observations are consistent with the findings of the necrosis observations, supporting the antiproliferative potential of the bacterial extract towards the MCF-7 cells.

## 4. Discussion

The capacity of marine microorganisms to create a diverse variety of bioactive metabolites demonstrates a key role in tackling several humanity-threatening challenges. Microbial metabolites may serve as antibacterial agents against a broad range of pathogenic microorganisms that have acquired resistance to current medications [56]. Furthermore, unlike conventional chemotherapeutics, unique natural chemicals may function as an effective antitumor medication that only kills cancer cells with minimal side effects on healthy ones [9]. For this study, different sediment samples were collected from various hypersaline environments in Egypt. One of the halotolerant isolates demonstrated potent antimicrobial activity against four pathogenic bacteria. The isolate was identified as *B. laterosporus* strain BF202. The first tool for the strain identification was to optimize the cultural conditions. Starting with the fermentation conditions, the maximum antibacterial activity of *B. laterosporus* filtrate was detected using a glucose peptone medium. The same method was applied by Usta et al. [57], who isolated *B. laterosporus* from various saline environments, including Paradip port in Odisha, India, and named *B. laterosporus* [58]. The bacterial filtrate was incubated for 18 h with continuous shaking, which is in contrast to [57], who recommended 28 h of incubation. On the other hand, mannose was the most used carbon source in our study owing to the superior antibacterial activity noticed in comparison to glucose and sucrose. In line with our observations, the results of Ak et al. [57] confirmed that mannose contributed to the maximum impact of *B. laterosporus*.

Ammonium carbonate outperformed other nitrogen sources in our study, as observed by the increase in *B. laterosporus* antibacterial activity against *S. aureus*. Ammonium is the primary nitrogen source that all microorganisms can directly use due to its easy absorption and metabolism [59]. Remarkable antibiotic activity of *B. laterosporus* ST-1 was also observed in cultures using glucose broth with yeast extract [59]. In another study, malt extract was recommended as the best nitrogen source, but the study doubted the role of yeast extract on culture [60]. Two other critical factors to be adjusted are the pH and temperature in order to balance the enzyme activity, oxidative-reductive reactions, and, most importantly, cellular membrane morphology. Among the pH values investigated, an initial pH of 7.0 resulted in the highest antibacterial activity of *B. laterosporus*, while a pH of 4.0 resulted in the lowest. This result was consistent with the outcomes of Usta et al. [57], who described the most significant antibacterial activity secondary to the application of pH 7.0. In parallel, 35 °C was the optimal temperature, in agreement with the previously reported data [57]. 

Five metabolites of *B. laterosporus* methanol extract were predicted by the GNPS molecular networking database in correlation to the proposed antimicrobial and antitumor activities, and classified as diketopiperazines. Diketopiperazines are the smallest known cyclic peptides, generally biosynthesized from amino acids. Moreover, diketopiperazines are produced by Gram-positive bacteria, fungi, and higher organisms, including mammals [61]. These groups of compounds have been evaluated for their antimicrobial, insecticidal, vasodilator, anti-tuberculosis, antioxidant, anti-viral, anti-prion, anti-hyperglycemic, and cytotoxic activities to demonstrate their potential applications in food, medicine, and agriculture [62]. Cyclo (Val-Phe), for instance, contributes to the antifungal properties of *Lactobacillus plantarum* [63], Cyclo (Ile-Tyr) of *Pseudomonas rhizosphaerae* exhibits antibacterial and antilarval potentials [64], and Cyclo (Phe-Pro) is claimed to induce apoptosis and thus to be effective as an antitumor molecule [65,66,67]. To the best of our knowledge, Cyclo (Val-Phe), Cyclo (Ile-Tyr), and Cyclo (Phe-Pro) are identified for the first time in this species. Quorum sensing (QS) facilitates communication processes among bacterial organisms, mediated by small, diffusible signals known as autoinducers and dependent on bacterial population density. Bacteria use QS to synchronize various functions, such as biofilm formation and pathogenicity. Quorum sensing is driven by autoinducer molecules, such as N-acyl-homoserine lactones produced by Gram-negative bacteria and oligopeptides-producing Gram-positive bacteria [68]. Diketopiperazines have interrupted the LuxR-mediated bacterial quorum-sensing systems, thus interfering with cell–cell communication [61]. As a result, diketopiperazines provide a new mechanism of action as anti-microbial agents manifested in the prevention of biofilm formation [61]. These results are in line with the findings published by [64,69,70,71], illustrating the antibacterial and antifungal properties of similar Cyclo peptides.

Interestingly, bacterial broth filtrate showed a potent cytotoxic effect on cancer cells (MCF-7) (IC_50_ = 7.93 µg/mL) and was harmless to normal cells. It is widely documented that apoptosis is a crucial factor in tumor regression, and ablation enhances tumor development [72]. 

In this vein, diketopiperazine compounds were proven effective as antioxidant and cytotoxic entities, supporting the antiproliferative potential against MCF-7 cells in this study [62]. The possible mechanisms of action have been discussed previously [73] and explained by foraging the intracellular ROS, such as superoxide, hydroxyl radicals, and hydrogen peroxide, besides diminishing the cytotoxicity and genotoxicity of the normal cells. The selective action was also reported by [73], documenting the antitumor activity of diketopiperazine Cyclo(L-Pro-D-Arg) against HeLa cells with an IC_50_ of 50 μg/mL while displaying no cytotoxicity against normal monkey kidney cells (Vero) using a concentration of up to 100 μg/mL. All in all, the data indicate that diketopiperazines extracted from *B. laterosporus* have a potent impact against drug-resistant germs and cancerous cells. 

## 5. Conclusions

The discovery of new bioactive compounds is a cornerstone for developing new pharmaceuticals. This study succeeds in determining the antibacterial and anticancer characteristics of the *B. laterosporus* broth filtrate. The ideal culture conditions for achieving the best antibiosis potential were first characterized prior to testing the metabolites on the MCF-7 cell lines. The *B. laterosporus* metabolites, namely diketopiperazines, were identified employing the LC-MS-MS data, and the antimicrobial and anticancer biological properties were confirmed both in vitro and by employing the molecular networking database. The results raise awareness of the importance of the evolved natural products including the halophilic dwelling bacteria of the Egyptian hypersaline habitats and thus invite scrutiny of their bioactivities. The findings highlight new approaches to investigate and explore uncommon natural resources with the hope of introducing new entities to the medical and pharmaceutical fields. 

## Figures and Tables

**Table 1:** Antibacterial activities of the isolated strains against the pathogenic bacteria using the paper disc method.

Microbial Isolates	Inhibition Zone Diameters			
	*E. coli*	*S. typhi*	*S. aureus*	*K. pneumoniae*
1	9 ± 2.64 ^c^	11 ± 4.58 ^bc^	7 ± 1.73 ^d^	10 ± 3.46 ^bc^
2	-	16 ± 2.64 ^ab^	12 ± 3.00 ^bcd^	17 ± 2.00 ^ab^
3	-	7 ± 1.00 ^cd^	-	9 ± 1.73 ^c^
4	11 ± 1.00 ^bc^	15 ± 2.64 ^abc^	-	12 ± 2.00 ^abc^
5	21 ± 2.64 ^a^	20 ± 7.00 ^a^	20 ± 4.35 ^a^	19 ± 2.64 ^a^
6	15 ± 2.64 ^abc^	11 ± 2.64 ^bc^	-	-
7	-	-	-	12 ± 2.64 ^abc^
8	9 ± 1.00 ^c^	12 ± 1.73 ^abc^	-	-
9	-	-	11 ± 1.30 ^cd^	14 ± 3.30 ^abc^
10	-	-	-	-
11	11 ± 1.73 ^bc^	13 ± 3.00 ^abc^	10 ± 1.00 ^cd^	9 ± 1.73 ^c^
12	-	-	14 ± 4.35 ^abc^	12 ± 1.00 ^abc^
13	10 ± 4.35 ^bc^	-	-	12 ± 3.46 ^abc^
14	-	14 ± 2.00 ^abc^	10 ± 2.64 ^cd^	11 ± 2.64 ^bc^
15	15 ± 4.35 ^abc^	13 ± 4.35 ^abc^	-	-
16	16 ± 4.35 ^ab^	13 ± 3.60 ^abc^	15 ± 2.64 ^abc^	16 ± 4.35 ^abc^
17	-	-	15 ± 2.64 ^abc^	10 ± 1.00 ^bc^
18	11 ± 1.73 ^bc^	15 ± 2.64 ^abc^	-	-
19	-	-	18 ± 4.35 ^ab^	12 ± 3.46 ^abc^
20	19 ± 1.00 ^a^	17 ± 2.64 ^ab^	-	14 ± 2.64 ^abc^

All values are presented as mean ± SD, (*n* = 3). The significance among growth inhibition zone diameters of each microbial isolate’s filtrate of the four selected pathogens was measured by Tukey’s HSD. Different letters represent significance, *p* < 0.05.

**Table 2:** Activity of cell culture supernatant of *B. laterosporus* BF202.

Pathogens	Inhibition Zone Diameters (mm)	
	Bacterial Filtrate	Levofloxacin
*E. coli*	23 ± 1.00 ^b^	33 ± 2.64 ^a^
*S. typhi*	22 ± 2.64 ^b^	34 ± 3.60 ^a^
*S. aureus*	20 ± 2.64 ^b^	31 ± 2.64 ^a^
*K. pneumoniae*	19 ± 2.64 ^b^	32 ± 1.00 ^a^

All values are presented as mean ± SD, (*n* = 3) and the significance among growth inhibition zone diameters was measured by Tukey’s HSD. Different letters represent significance, *p* < 0.05.

**Table 3:** List of the identified metabolites, parent masses, and their fragments from the raw mass spectrum compared with that of the molecular networking database.

Compound Name	Parent Mass *m/z* (g/mol)	Adduct	Raw Material Fragments	Literature Fragment	Structure	Source
Cyclo (Leu-Pro) 1	211.257	[M + H]^+^	183.37, 155.17, 138.19, 86.14 and 70.11	154.1, 138.1, 125.1, 110.1, 98.1, 84.1, and 70.1 [53]	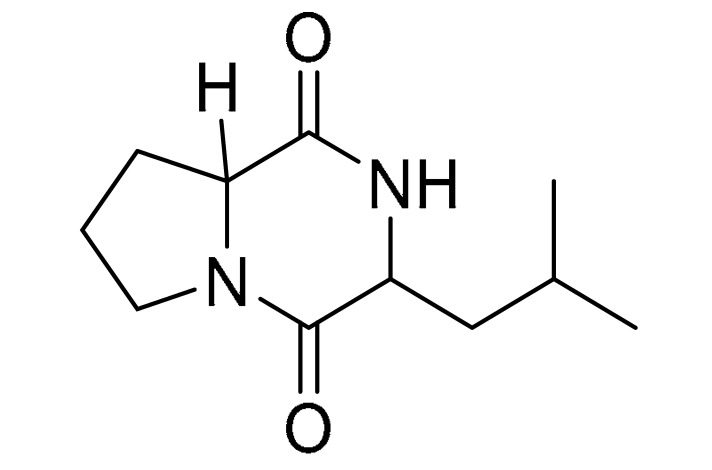	*Brevibacillus laterosporus-* *Bacillus* sp.
Cyclo-(Val-Phe) 2	245.24	[M-H]^−^	219.16, 202.13 and 120.08	219, 202, 174, 157, and 120 [54]	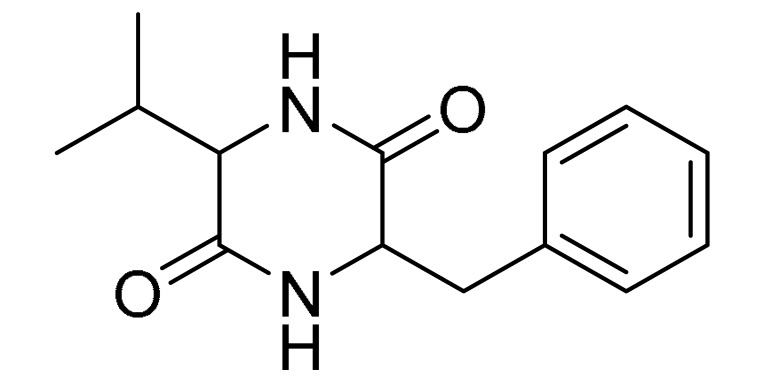	Endophytic *Streptomyces*
Cyclo (Tyr-Pro) 3	261.188	[M + H] ^+^	233.13, 155.08, 147.04 and 136.08	NA	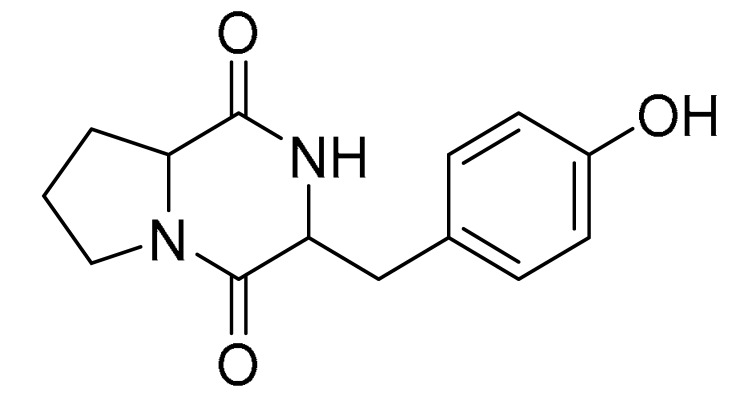	*Bacillus* sp.
Cyclo (Ile-Tyr) 4	277.29	[M + H] ^+^	249.16, 232.13, 171.11 and 136.08	249, 232, 204,171, and 136 [54]	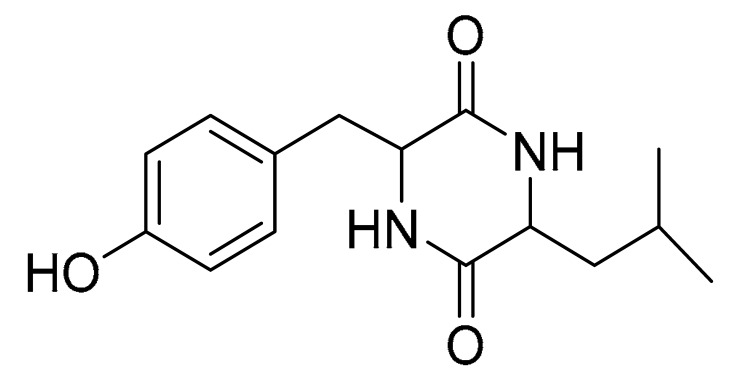	Marine-Derived Bacteria Harizani
Cyclo (Phe-Pro) 5	245.131	[M + H] ^+^	120.09 and 70.07	217, 200, 172, 154, 120, and 70 [54]	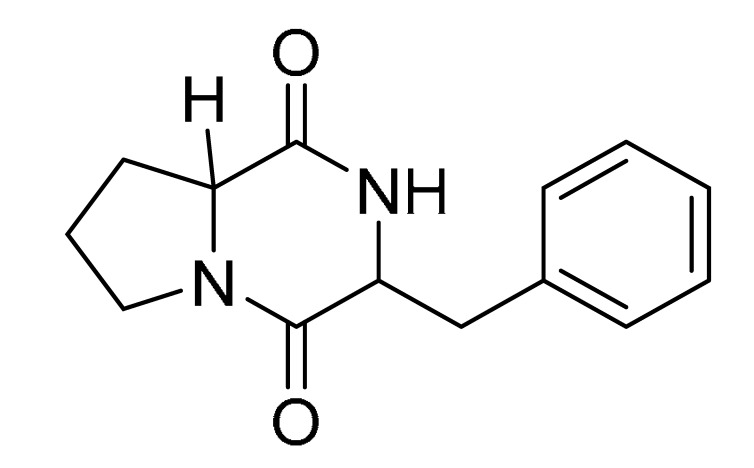	Endophytic *Streptomyces*
Tyr-Pro 6	279.255	[M + H] ^+^	136.08 and 116.07	147.04, 142.95, 137.07, 136.07, 121.05, 119.04, 118.06, 116.06, 109.06, 92.05, 71.06, and 70.06 [55]	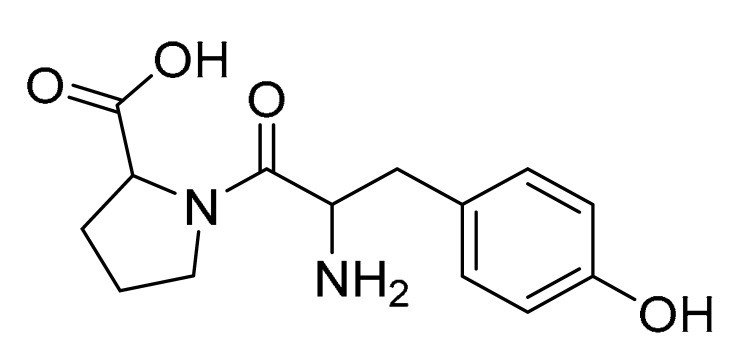	Marine-Derived Bacteria
Cholic acid 7	374.454	[M + H-2H_2_O] ^+^	355.21 and 337.19	N/A	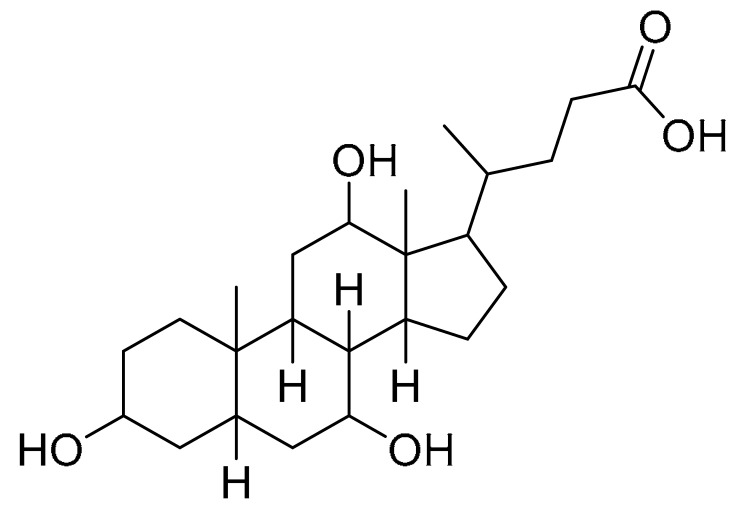	Fecal Bacteria from Cholesterol Gallstone Patients Marine Ascidian-Associated Bacterium *Hasllibacter halocynthiae*

## Data Availability

Not applicable.
